# In-depth analysis of erythrose reductase homologs in *Yarrowia lipolytica*

**DOI:** 10.1038/s41598-023-36152-x

**Published:** 2023-06-05

**Authors:** Mateusz Szczepańczyk, Dorota A. Rzechonek, Cécile Neuvéglise, Aleksandra M. Mirończuk

**Affiliations:** 1 Laboratory for Biosustainability, Institute of Environmental Biology, Wrocław University of Environmental and Life Sciences, 5B Kozuchowska St., 51-631 Wroclaw, Poland; 2 INRAE, Institut Agro, SPO, University Montpellier, 34060 Montpellier, France

**Keywords:** Biotechnology, Microbiology

## Abstract

The unconventional yeast *Yarrowia lipolytica* produces erythritol as an osmoprotectant to adapt to osmotic stress. In this study, the array of putative erythrose reductases, responsible for the conversion of d-erythrose to erythritol, was analyzed. Single knockout and multiple knockout strains were tested for their ability to produce polyols in osmotic stress conditions. Lack of six of the reductase genes does not affect erythritol significantly, as the production of this polyol is comparable to the control strain. Deletion of eight of the homologous erythrose reductase genes resulted in a 91% decrease in erythritol synthesis, a 53% increase in mannitol synthesis, and an almost 8-fold increase in arabitol synthesis as compared to the control strain. Additionally, the utilization of glycerol was impaired in the media with induced higher osmotic pressure. The results of this research may shed new light on the production of arabitol and mannitol from glycerol by *Y. lipolytica* and help to develop strategies for further modification in polyol pathways in these microorganisms.

## Introduction

The constant growth of the human population leads to higher demands for food production and the invention of alternative sources for many chemical compounds currently obtained from natural components or by chemical synthesis. The usage of microorganisms to produce valuable products remains one of the best ways to deal with the increase in demand for those products^1–3^. To obtain the best production parameters, the culture conditions and the media are also constantly under investigation. For economical reasons, it is best when the microorganisms can utilize waste materials, such as by-products of large industrial processes, including lignocellulosic biomass^4^ and crude glycerol^5–7^. All these features make the non-conventional yeast *Yarrowia lipolytica* a suitable host for many industrial applications. This yeast possesses the ability to utilize many unconventional carbon sources and is characterized by high resistance to unfavorable conditions, such as osmotic stress^8,9^. Another advantage is the possibility of growth in media prepared with the addition of seawater instead of drinking water^10^.

The oleaginous yeast *Y. lipolytica* is a model organism for numerous processes, such as lipid metabolism^11–14^ and polyol synthesis^15–18^ and well as acting as a host for heterologous protein synthesis^19–21^. This yeast possesses the ability to withstand unfavorable conditions such as low and high pH and high osmotic pressure. Yeasts from the *Yarrowia* clade were isolated from seas and water reservoirs, which also contain high concentrations of salts, inducing osmotic stress. Well-established methods for genetic modifications of this organism led to the heterologous expression of genes involved in the synthesis of many desired products, such as β-carotene^22^, α-farnesane^23^ and pentane^24^. The biotechnological procedures with this yeast species obtained the GRAS (Generally Recognized as Safe) status awarded by the US Food and Drug Administration. The biomass produced from crude glycerol was allowed to be used as an addition to fodder^25^. However, one of the best-studied processes involving *Y. lipolytica* remains polyol synthesis. This organism can produce erythritol, arabitol and mannitol simultaneously^16^. Production of erythritol occurs as a response to osmotic stress that can be obtained by a high concentration of glycerol, which is a possible carbon source for *Y. lipolytica*, allowing the conversion of glycerol to erythritol^26^. Additionally, different yeasts belonging to the *Yarrowia* clade have also been investigated as potential hosts for polyol production from pure and crude glycerol^27,28^.

The conditions required for the production of polyols are already established and well researched^15,29^, but the molecular mechanism behind those pathways remains under investigation. It is due to the intermingled web of dependencies in the production of erythritol, arabitol and mannitol^17,30^. Some of the proteins seem to be necessary for the synthesis of more than one polyol, while others, responsible for the synthesis of one polyol, may affect the utilization of different polyols. The multiple roles of proteins in polyols metabolism are an important issue due to the possibility of synthesis of all the mentioned polyols simultaneously. This impacts the production parameters and blocks the ability to utilize *Y. lipolytica* as a host for the production of mannitol and arabitol on an industrial scale, as erythritol remains the most prevalently produced polyol^30^. Resolving the issue of shifting carbon flux from erythritol to other polyols may result in obtaining new potential hosts for microbial synthesis of arabitol and mannitol from waste materials.

As the previous studies did not identify which erythrose reductase (ER) is the primary gene in the process^30^ and the lack of one of the genes did not affect the production of erythritol significantly^18,31^, the study aimed to analyze the entire group of highly homologous erythrose reductases. To accomplish this, multiple knockout strains were obtained and their ability to produce erythritol, as well as mannitol and arabitol, was analyzed. Those results shine a new light on the importance of the ER homologs and their overall impact on polyol synthesis and osmoprotection. Homologs of *Y. lipolytica* erythrose reductases (YlERs) were also found of the species of the *Yarrowia* clade. The potential for polyol synthesis by all of the species was analyzed^27^ but none of them had better production parameters than *Y. lipolytica*. Polyol synthesis in the clade was additionally investigated in terms of genetic differences in the number of copies of ER homologs. Analysis of the evolutionary scenario of erythrose reductase genes was performed to further explore the phenomenon regarding the number of homologs in yeast belonging to the *Yarrowia* clade.

An issue complicating the understanding of polyol synthesis is the presence of multiple homologs of the genes in the genome of *Y. lipolytica*^32,33^. One example is erythrose reductase (ER), responsible for the conversion of d-erythrose to erythritol. This reaction is carried out with NADPH as an electron donor and the enzyme responsible for catalyzing this process belongs to the aldo–keto reductase family^31^. The first reported YlER was described with a group of seven other highly similar homologs, characterized by similarity to previously reported enzymes in *Candida magnoliae* and *Moniliella megachiliensis*^18^. In silico analysis revealed that the genes encoding the reductases are present on all six of the chromosomes of *Y. lipolytica* (Fig. S1). The research into the process of erythritol synthesis continued. New potential reductases were described and their impact on polyol production was analyzed^31^.

## Results

### In silico analysis of the *Yarrowia* clade

**Figure 1:**
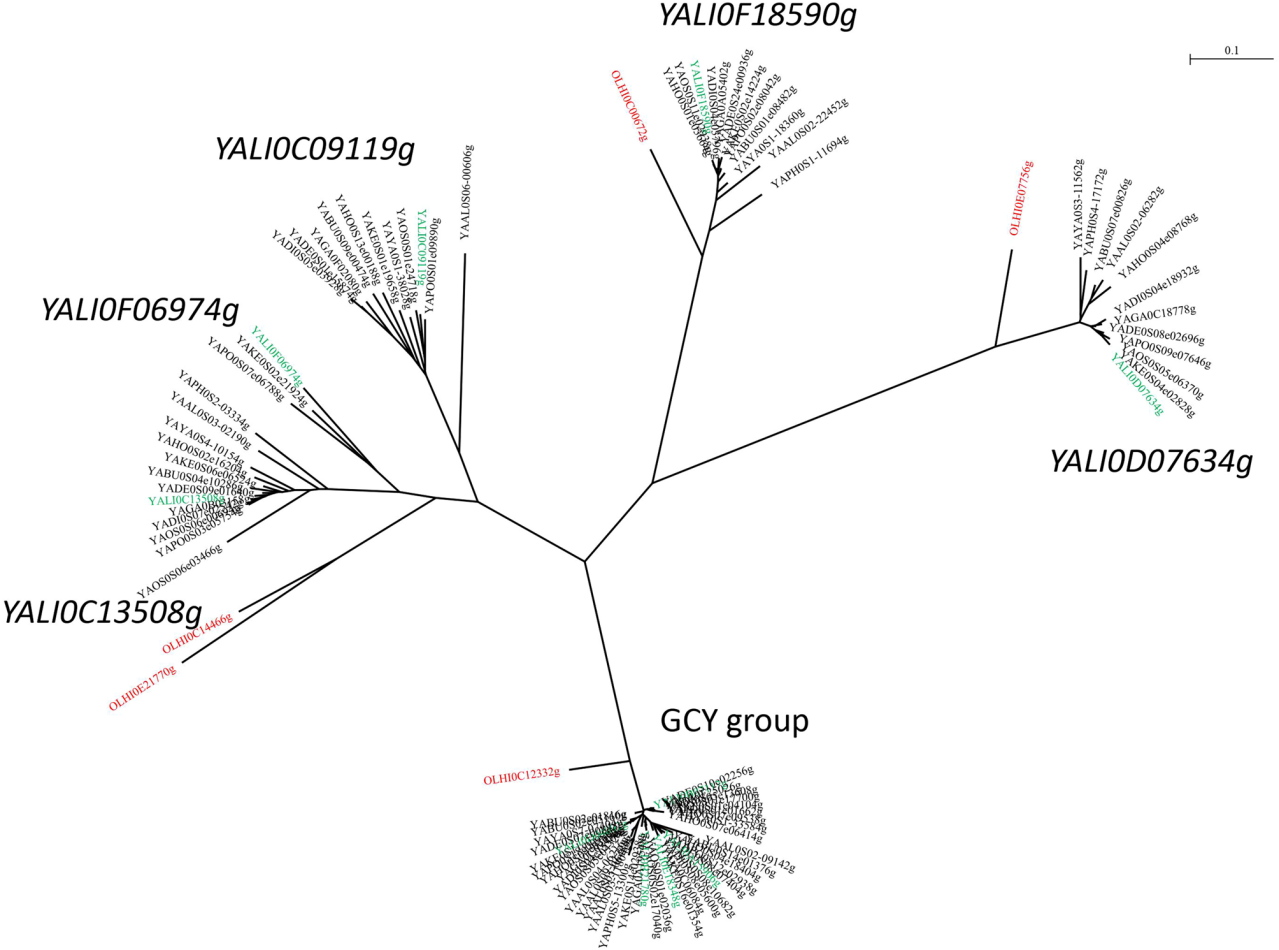
Phylogenic tree of the aldo/keto reductases in the *Yarrowia* clade. *Y. lipolytica* genes are in green and those of *Candida hispaniensis* in red. ARA12–*YALI0C13508g*, ARA1–*YALI0F06974g*, ARA 13–*YALI0C09119g*, GCY15–*YALI0F18590g*, GRE3–*YALI0D07634g*.

In the genome of *Y. lipolytica*, a group of eight highly homologous YlERs have been selected^18^. All the proteins are characterized by a high level of similarity, reaching an average homology of around 90%. In silico analysis of the selected genes revealed that YlERs which possesses the conserved aldo/keto reductase domain belonging to the GCY family (galactose-induced protein of aldo/keto reductase family): *YALI0B07117g* (GCY12), *YALI0A15906g* (GCY13), *YALI0B21780g* (GCY16); glycerol dehydrogenase of the aldo/keto reductase family: *YALI0D04092g (*GCY1), *YALI0E18348g* (GCY14), *YALI0F18590g* (GCY15); other proteins of the aldo/keto reductase family: *YALIC13508g (ARA12)* and *YALIC09119g* (ARA13). For further analysis, two additional genes were added, due to their potential role as erythrose reductases and a high level of similarity: *YALI0F06974g* (ARA1), which also belongs to the aldo/keto reductase family, and *YALI0D07634g* (GRE3), characterized as aldose reductase. The phylogenic tree of the genes in the *Yarrowia* clade is represented in Fig. 1.

The most conserved group of YlERs contains the genes belonging to the GCY family. Due to their high similarity a closer examination has been carried out on their role and occurrence in other species belonging to the *Yarrowia* clade (Table 1).

**Table 1:** Number of orthologous GCY genes in *Yarrowia* clade species.

	*YALI0* *D04092g*	*YALI0* *B07117g*	*YALI0* *A15906g*	*YALI0* *E18348g*	*YALI0* *B21780g*	Other ER	Total per strain
*Y. alimentaria*	**1**	**1**	*0*	*0*	**1**	***2***	5
*Y. bubula*	*0*	**1**	**1**	*0*	**1**	*0*	3
*Y. deformans*	**1**	**1**	**1**	*0*	*0*	*0*	3
*Y. divulgata*	**1**	**1**	**1**	*0*	*0*	*0*	3
*Y. galli*	**1**	**1**	**1**	*0*	**1**	*0*	4
*Y. hollandica*	**1**	**1**	**1**	*0*	**1**	*0*	4
*Y. keelungensis*	**1**	**1**	**1**	*0*	**1**	*0*	4
*Y. lipolytica*	**1**	**1**	**1**	**1**	**1**	*0*	5
*Y. osloensis*	**1**	**1**	**1**	*0*	**1**	**1**	5
*Y. phangngaensis*	*0*	**1**	*0*	*0*	*0*	*0*	1
*Y. porcina*	**1**	**1**	**1**	*0*	*0*	*0*	3
*Y. yakushimensis*	*0*	**1**	*0*	*0*	**1**	*0*	2
*C. hispaniensis*	*0*	*0*	*0*	*0*	*0*	**1**	1
Total per GCY	9	12	9	1	8	4	43

Significant values are in [bold, italics, bolditalic].

### Analysis of the strains growth

**Figure 2:**
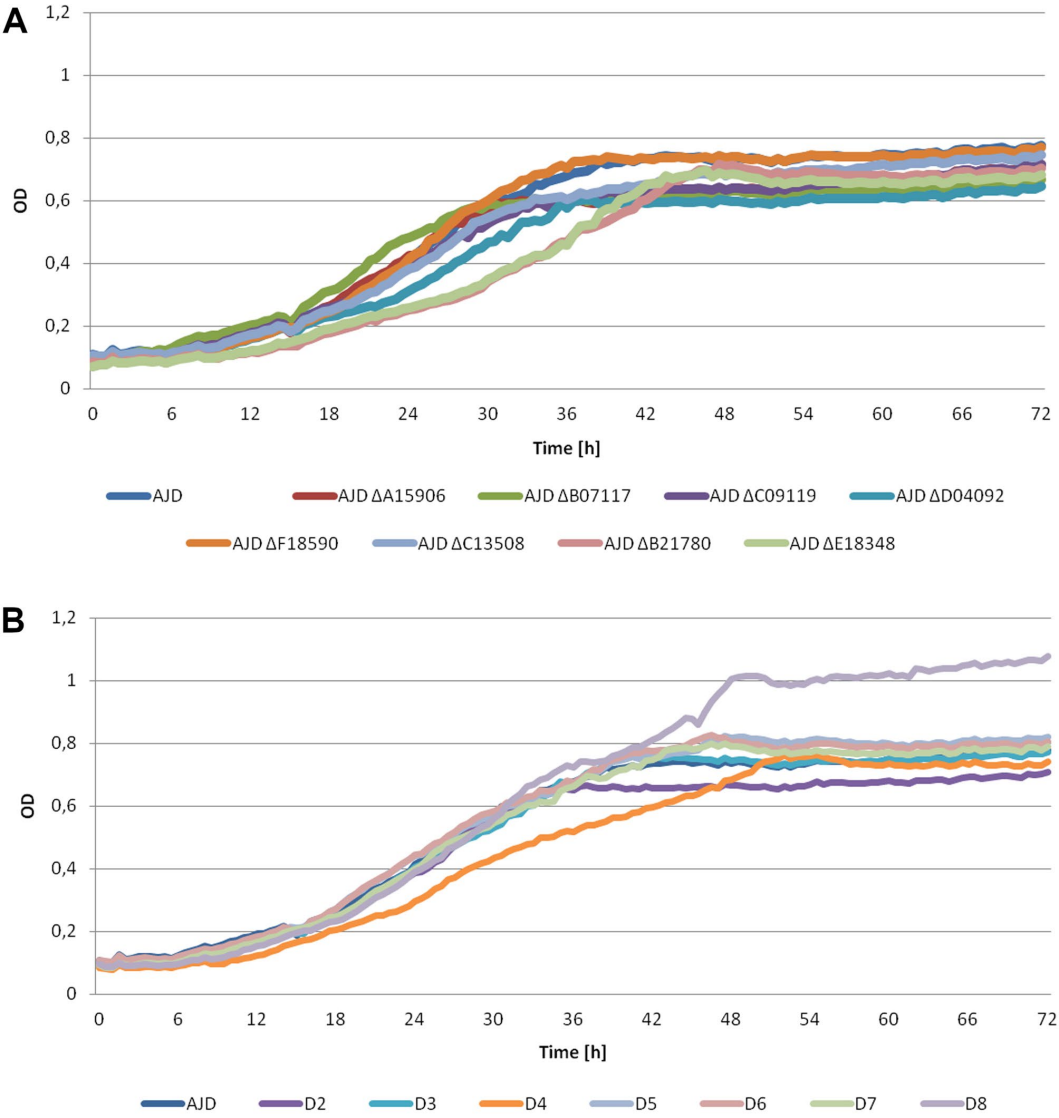
Growth analysis of all the obtained strains as compared to the control strain (AJD) in YNB medium with supplementation of glycerol (5%). The optical density at the beginning of the experiment was set at 0.1. The analysis lasted 72 h. (**A**) The single knockout mutants and the control strain; (**B**) Multiple knockout strains and the control strain.

To investigate the role and impact of selected YlERs, multiple knockout strains were obtained in the *Y. lipolytica* AJD strain and tested in the laboratory setting (Table 2). First, the viability of each created strain was determined, to eliminate the possible effect of impaired growth on polyol production in the later stages of the experiment. This analysis was performed on four different media (YPD, YBN with 5% glucose (w/v), 5% glycerol (w/v) or 10% glycerol (w/v)) with both glycerol and glucose as sole carbon sources. A higher concentration of glycerol was tested due to the components of the erythritol synthesis medium used for the analysis of the polyol production profile. The results, shown in Fig. 2, prove that the genetic modifications of the strains did not affect their growth.

**Table 2:** The multiple knockout strains created in this study and their names.

Gene name	Strain name						
	D2	D3	D4	D5	D6	D7	D8
*YALI0E18348g*	✔	✔	✔	✔	✔	✔	Δ
***YALI0B21780g***	✔	✔	✔	✔	✔	Δ	Δ
*YALI0C13508g*	✔	✔	✔	✔	Δ	Δ	Δ
***YALI0D04092g***	✔	✔	✔	Δ	Δ	Δ	Δ
***YALI0A15906g***	✔	✔	Δ	Δ	Δ	Δ	Δ
*YALI0F18590g*	✔	Δ	Δ	Δ	Δ	Δ	Δ
*YALI0C09119g*	Δ	Δ	Δ	Δ	Δ	Δ	Δ
***YALI0B07117g***	Δ	Δ	Δ	Δ	Δ	Δ	Δ

The initial knockout was carried out in *Y. lipolytica* strain AJD. Δ describes which gene was deleted and ✔ represents the intact genes. Genes belonging to the GCY group are in bold.

The multiple knockout strains grew at a similar level to all the single knockout strains and the control strain (AJD) reached the optical density of 0.6–0.8. The D8 mutant showed improved growth on the YNB medium with 5% glycerol, contrary to the expected result. This feature was also visible in the YNB medium with 5% glucose (data not shown) in which both the D8 and D7 strains grew to a slightly higher OD value than the rest of the strains in spite of the multiple consecutive knockouts. Improved growth was not observed in the YNB medium with an increased concentration of glycerol (10% w/v). All of the analyzed strains did not differ in growth rates on the YPD medium, obtaining an OD of 1.2 (data not shown).

### Impact of ERs knockouts on glycerol utilization

As the strains showed similar growth in all the analyzed media. The experiment was scaled up to 0.25 L baffled shake flasks with 30 mL of erythritol synthesis medium (ESM), containing 10% glycerol (w/v). This concentration of glycerol was tested in the growth experiment and did not impair the growth of any of the constructed strains despite the multiple genetic modifications.

**Figure 3:**
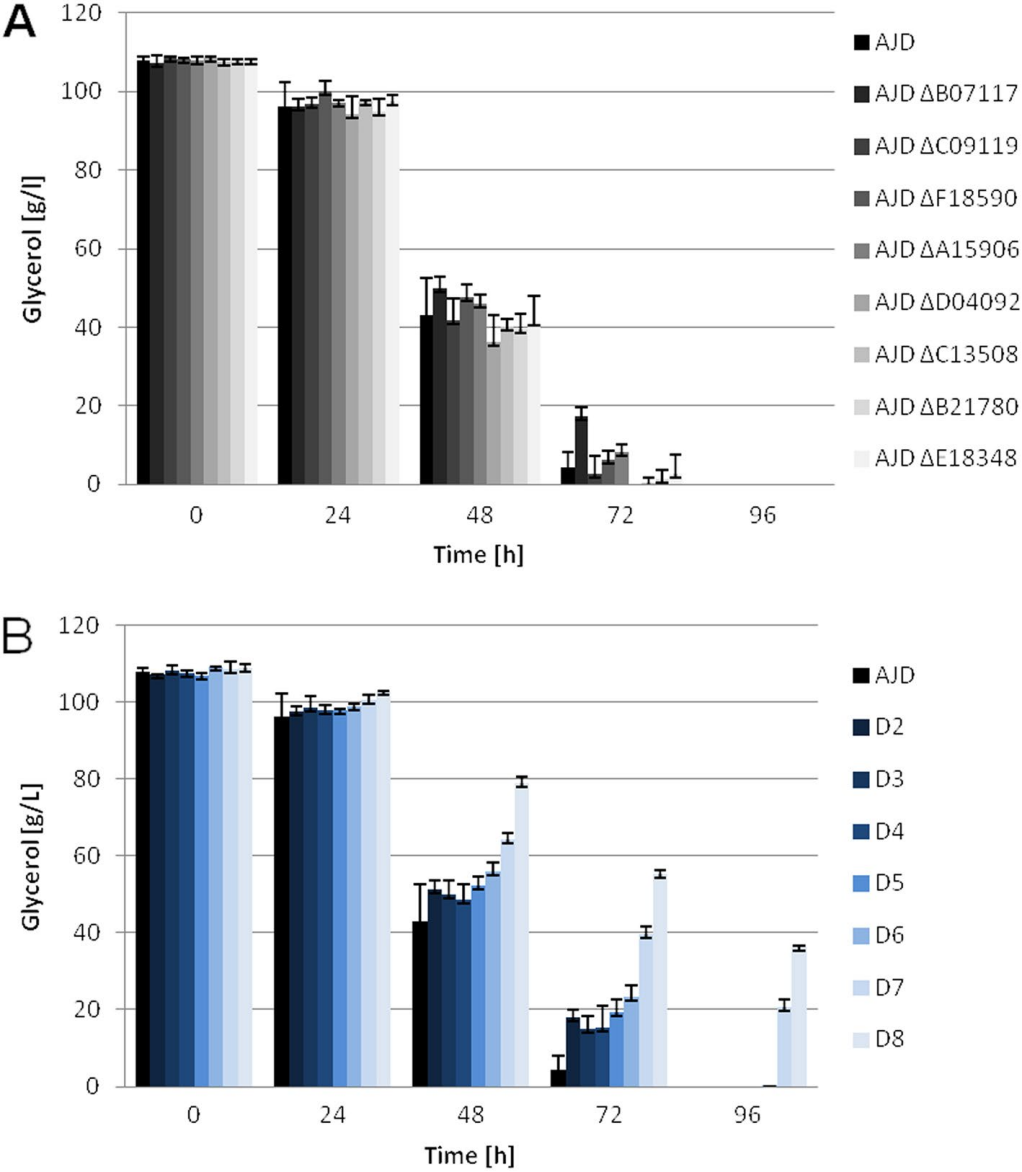
Glycerol utilization in shake flask experiment with ESM containing glycerol (110 g/L) as the main carbon source. (**A**) control strain and single knockout strains; (**B**) control strain and multiple knockout strains.

The first step of the study was the analysis of the impact of lacking one or multiple genes of erythrose reductases on polyol production in the shake flask experiments with ESM. Firstly, the analysis focused on the utilization of glycerol from the media. As shown in Fig. 3, the strain with the knockout of *YALI0B07117g* is characterized by the slowest utilization rates out of all the single knockout strains. The detected concentration of glycerol for this strain after 72 h of cultivation was 17.26 (± 2.52) g/L while the value at the same time point for the control strain was 4.24 (± 3.87) g/L, similarly to the rest of the group of strains with single knockout.

In contrast to the previous experiment, a delay in glycerol utilization for the multiple knockouts strains was observed. The glycerol uptake decreases significantly in the strains with seven (D7) and eight (D8) knockouts. After 96 h of cultivation, neither strain depleted the glycerol completely. Residual glycerol was also observed in the culture with the D6 strain. The concentration of glycerol after 96 h of cultivation was 20.51 (± 2.3) g/L of glycerol for the D7 strain and 36.32 (± 0.39) g/L for the D8 strain. The rate of glycerol utilization dropped drastically from 1.44 g/L/h for the control strain after 72 h of cultivation, where glycerol was still present, to 0.96 g/L/h and 0.74 g/L/h for D7 and D8 strains, respectively. The utilization of glycerol did not change significantly in the strains with single knockouts except for AJD ΔB07117, which shows a similar trend to the multiple knockout strains (D2-D6). This result may indicate the role of *YALI0B07117g* in glycerol uptake as well as its importance for the erythritol synthesis overall. That is also visible in the polyol production profile, where the shift to arabitol and mannitol is observed. Additionally, the impairment with glycerol uptake could be affected by the limited erythritol production by the strain in the conditions evoking the osmotic stress response.

### Analysis of impact of the osmotic stress

**Figure 4:**
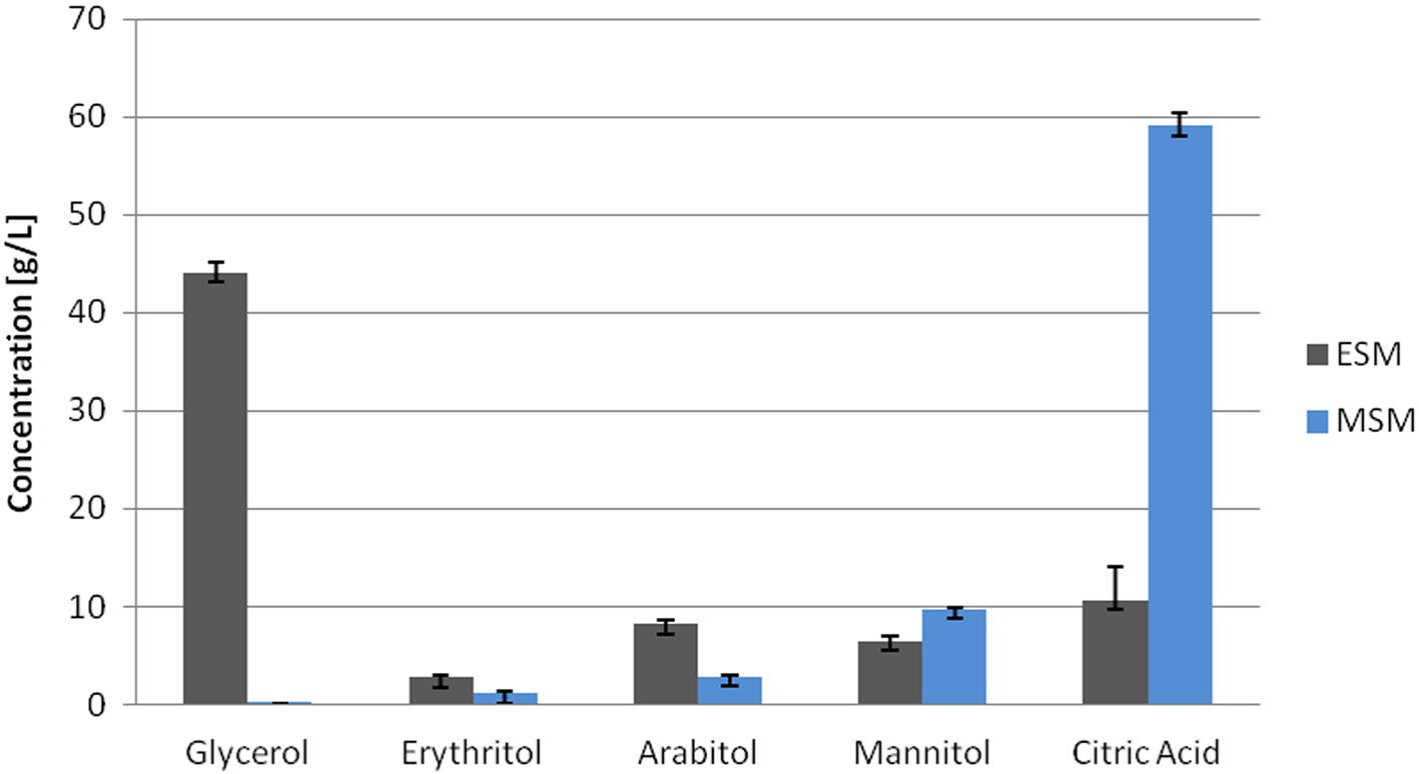
Glycerol utilization, polyols and citric acid production by D8 strain in both erythritol synthesis medium (ESM) (grey) and mannitol synthesis medium (MSM) (light blue), after 96 h of cultivation.

To verify the glycerol consumption rate by strain D8 an additional experiment with ESM was set up. The time of the experiment was dependent on the glycerol uptake. The strain utilized nearly all of the glycerol within 168 h, with 3.75 g/L of glycerol left in the medium (data not shown). The ERs are responsible for the synthesis of erythritol under osmotic stress; therefore the influence of salt added to the medium on the growth and glycerol utilization was compared with the parameters observed in the mannitol synthesis medium (MSM), which is a modified ESM lacking sodium chloride^29^. The results of this experiment for the D8 strain are shown in Fig. 4. The utilization of glycerol was impaired in the presence of salt, as its concentration was measured at 44.08 (± 1.0) g/L as compared to 0.02 (± 0.03) g/L in the MSM after 96 h of cultivation. Additionally, the production of citric acid was increased immensely for the strain in conditions without osmotic pressure, reaching close to 60 g/L. Both media were adjusted to pH 3.0 and supplemented with CaCO_3_ to maintain this value. Interestingly, the production of citric acid for wild type strains of *Y. lipolytica* is very low in those conditions^34,35^. A decrease in arabitol and erythritol production was also observed for the cultivation in mannitol synthesis medium. The results indicate the profound impact on the polyol production profile and glycerol utilization caused by the addition of salt to the medium, whereas the high concentration of glycerol did not have the same limiting effect on the D8 strain.

**Figure 5:**
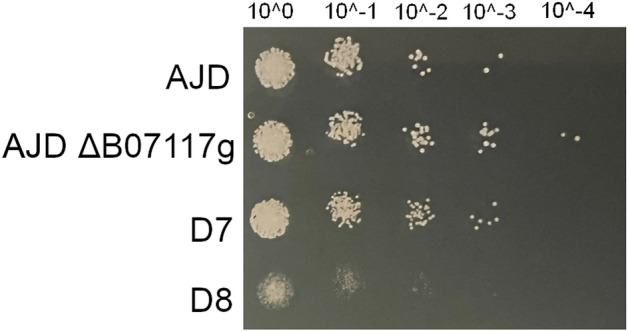
Spot-test analysis of the AJD, AJD ΔB07117g, D7 and D8 strains on YNB medium with 2% glycerol and addition of 1 M NaCl. The photo was taken after 72 h of growth at 28 °C. Initial OD was set at 0.2, successive dilutions are indicated on the top.

To confirm the cell response to osmotic stress, strain growth was analyzed on media plates containing salt. YNB media plates with 2% of glycerol were supplemented with NaCl solution to a total concentration of 0.25, 0.5, 0.75 and 1 M for the spot-test analysis. The osmotic stress impaired the growth of the D8 strain significantly in the last analyzed condition (Fig. 5). The lower concentrations did not affect the growth visibly.

### Effects of ER genes knockout on polyols production

**Figure 6:**
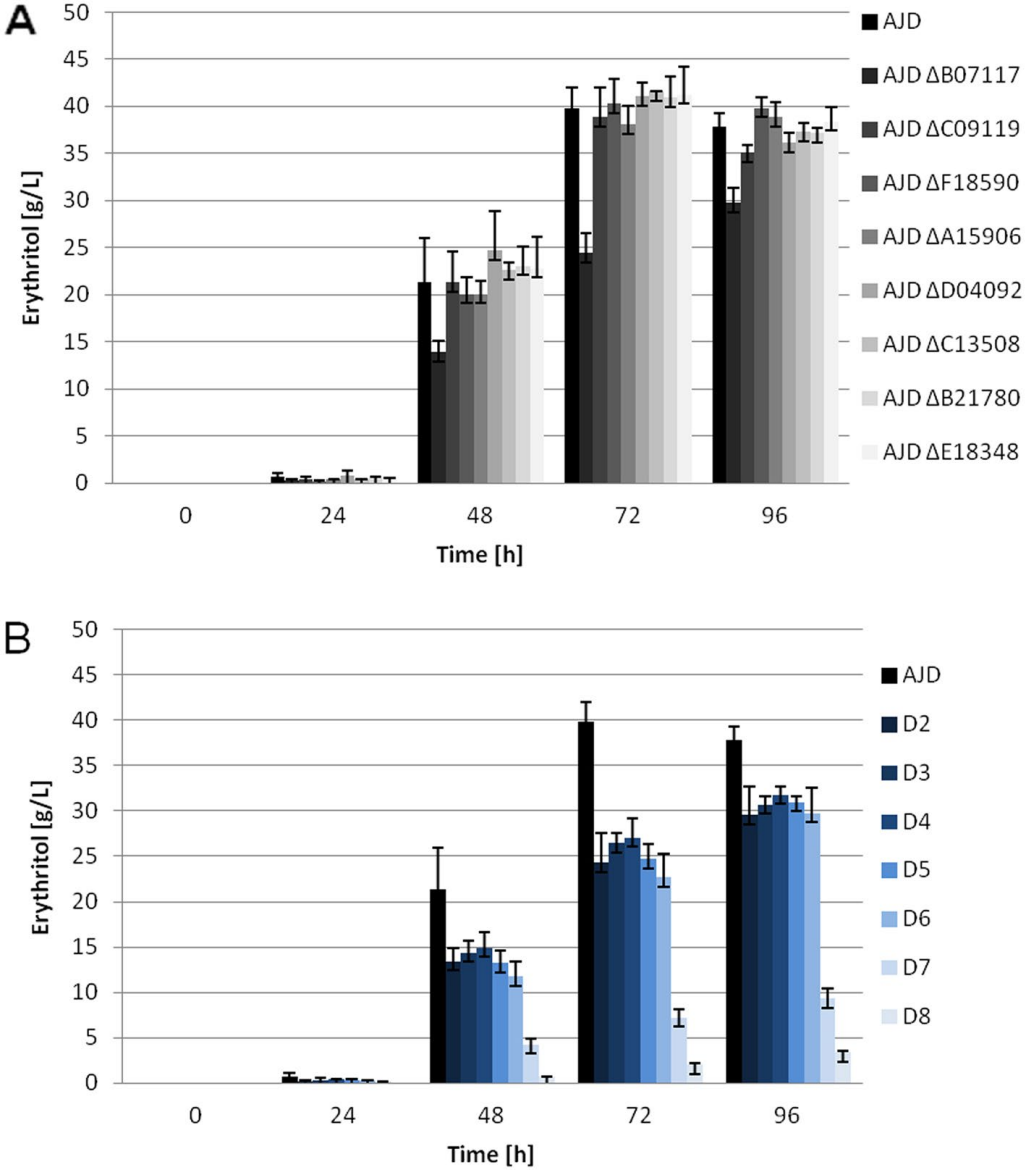
Erythritol synthesis in ESM. (**A**) control strain (black) and single knockout mutants (shades of black); (**B**) control strain (black) and multiple knockout mutants (shades of blue).

To confirm a key role of ER in biosynthesis of erythritol the synthesis of this compound was measured in ESM, as shown in Fig. 6. Interestingly, the most significant decrease of erythritol synthesis is observed for the AJD ΔB07117 strain in the group of single knockout mutants and D7 and D8, which produce around 25% and 9%, respectively, of the erythritol as compared to the control strain after 96 h of cultivation. Erythritol appears in the sample collected after 48 h of cultivation for the D8 strain. The role of *YALI0B07117g* in erythritol synthesis was described before^30^. The impact of the knockout of a single ER is limited except for *YALI0B07117g*. Observations show that the knockout of as many as six of the ERs from this homologous group does not impair the polyol production significantly.

Due to the previous analysis of YlER knockouts^18,30,31^, the impact of the modifications was also analyzed with respect to other polyols. The concentration of mannitol and arabitol was measured, due to the synthesis of these polyols in an ESM to obtain the full production profile of each mutant strain.

**Figure 7:**
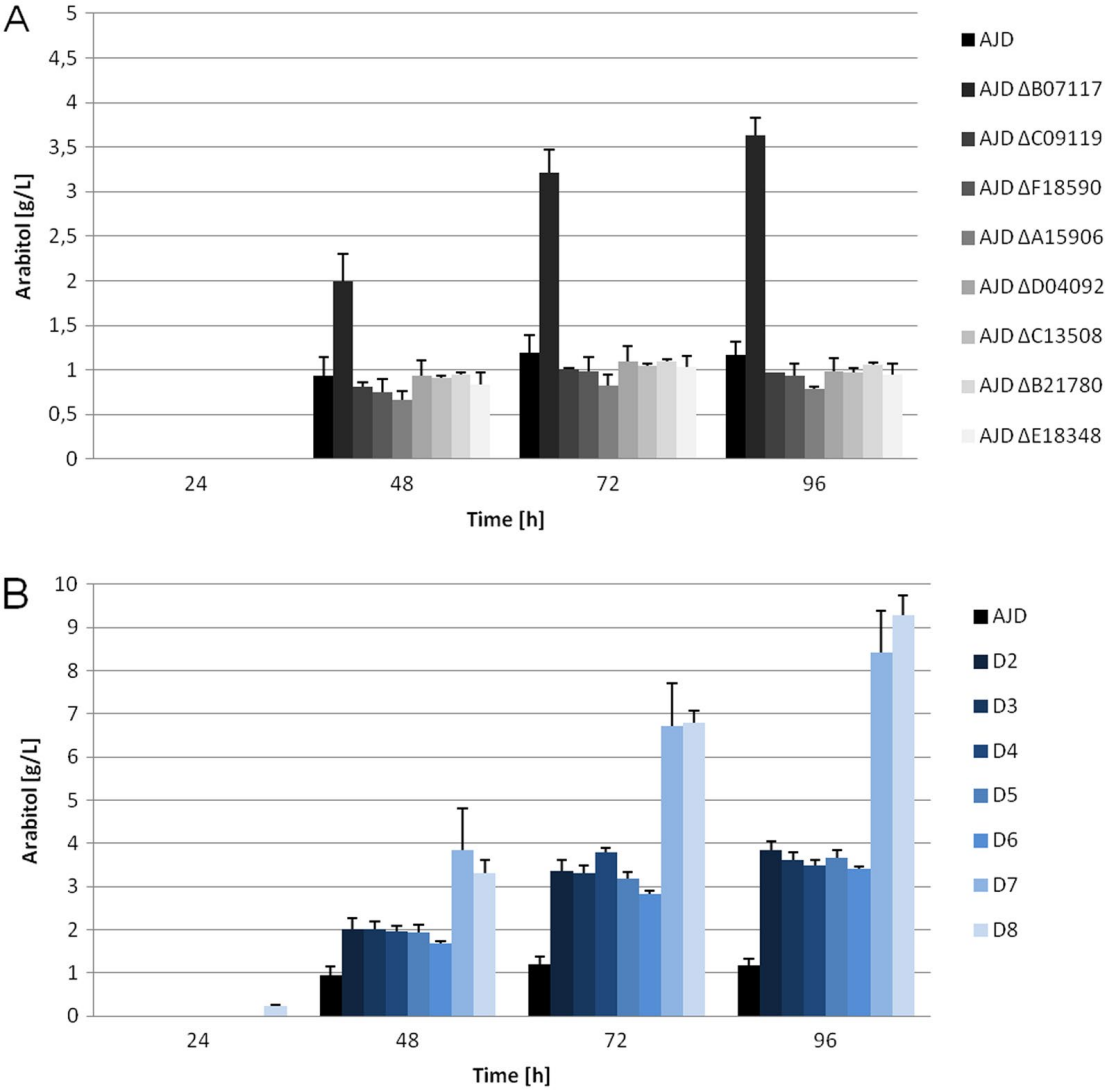
Arabitol synthesis in the shake flask experiment with ESM.

Concentration of arabitol and mannitol in medium was measured as both of those polyols are co-produced during erythritol synthesis. Knockout of the single erythrose reductase genes showed that there is a carbon flux towards mannitol and arabitol if the erythritol synthesis pathway is affected. The most striking example is the AJD ΔB07117 strain (Figs. S7 and 7). Significant differences were also observed in the group of multiple knockout strains. In strain D8 the synthesis of both mannitol (Fig. S2) and arabitol (Fig. 7) was detected earlier than the synthesis of erythritol.

The increase in mannitol production was observed for the AJD ΔB07117 strain, where the concentration of this polyol reached 6 g/L. This result was specific for this particular ER in the single knockout group. Considering the production patterns for other ER knockout strains it seems, that none of the genes except for *YALIB07117g* has a significant impact on mannitol synthesis. Since all of the multiple knockout strains had a knockout of the *YALIB07117g*, the fact that the production of mannitol was at or above the 6 g/L was not unexpected. Production of mannitol for the D7 strain was 77% higher as compared to the control strain (AJD) and 53% higher for the D8 strain, reaching 7.81 (± 0.61) g/L and 6.82 (± 0.49) g/L respectively (Fig. S2). Further analysis of arabitol concentration in the media followed that trend. Arabitol synthesis significantly improved for the AJD ΔB07117 strain and the multiple knockout strains. The values increased from 1.17 (± 0.15) g/L for the AJD strain to around 3.6 g/L for strains AJD ΔB07117 and D2-D6 strains. Production of arabitol reached 8.42 (± 0.94) g/L and 9.28 (± 0.46) g/L for strains D7 and D8, respectively, giving an 8 fold-improvement. The lack of other ER genes in the case of arabitol had an impact on the production of this polyol, contrary to production of mannitol.

**Figure 8:**
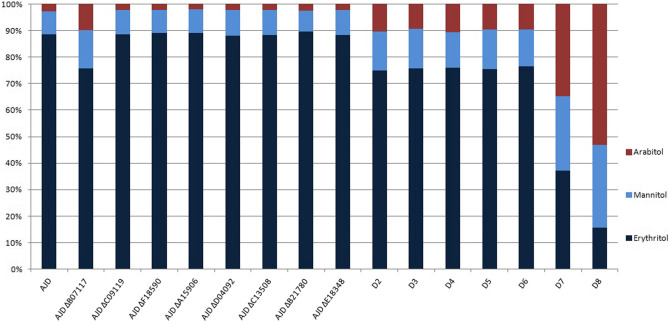
Production of polyols by the percentage they represent after 72 h of growth of the strains in an ESM before consumption by the cells.

Since production of polyols is a cellular response to high osmotic stress, the variation in the osmoprotectant pool was analyzed^9^. Erythritol is a major polyol produced by *Y. lipolytica* under high osmotic pressure. However, for strains with knockout of 7 or 8 homologs of ER, the profile of the synthesized polyols significantly shifted toward arabitol and mannitol. The results are summarized in Fig. 8. The holistic polyol production profile for the multiple knockout strains (D2-D6) is similar to the single knockout strain of the *YALI0B07117g* gene, suggesting that this homolog plays a key role in this process. Other single-knockout mutants do not differ from the control strain. Production of arabitol for the D8 strain exceeds 50% in the conditions optimized for erythritol synthesis.

### Biomass analysis post cultivation

Finally, the biomasses produced by the tested strains on ESM were analyzed as shown in Table 3. Interestingly, biomasses of the engineered strains were higher (except for AJD ΔB07117) than those of the control strain. These results confirm those previously obtained from microplate reader experiments, where the growth rate of all strains remained stable, without a negative influence of multiple gene deletions. The maximum value for biomass was measured for the D2 strain and reached 10 g/L. Additionally, parameters of yield and productivity of polyols and citric acid were calculated to provide more in depth analysis of the process and take into account the utilized glycerol and produced biomass. The results show the impact of *YALI0B07117g* on polyol production pattern and show similarities in the production parameters between the AJD ΔB07117 strain and multiple knockout strains (D2-D6), while all the rest of the single knockout mutants have parameters more closely related to the control strain. Strains D7 and D8 have distinctly higher parameters of yield and productivity for arabitol and lower for erythritol production.

**Table 3:** Biomass analysis (X), productivity (Q_ERY_) and yield (Y_ERY_) parameters for polyols and citric acid post cultivation in the ESM during shake-flask experiment after 96 h.

Strain	X g dm^-3^	Erythritol			Arabitol			Mannitol			Citric acid			Total polyols		
		ERY g dm^-3^	Y_ERY_ g g^-1^	Q_ERY_ g dm^-3^ h^-1^	ARA g dm^-3^	Y_ARA_ g g^-1^	Q_ARA_ g dm^-3^ h^-1^	MAN g dm^-3^	Y_MAN_ g g^-1^	Q_MAN_ g dm^-3^ h^-1^	CA g dm^-3^	Y_CA_ g g^-1^	Q_CA_ g dm^-3^ h^-1^	POLYOLS g dm^-3^	Y_POLOLS_ g g^-1^	Q_POLYOLS_ g dm^-3^ h^-1^
AJD	6.77	37.79	0.35	0.39	1.17	0.01	0.01	4.39	0.04	0.05	14.68	0.14	0.15	43.35	0.40	0.45
AJD ΔB07117	6.33	29.74	0.28	0.31	3.63	0.03	0.04	6.41	0.06	0.07	15.45	0.14	0.16	39.77	0.37	0.41
AJD ΔC09119	7.5	35.09	0.32	0.37	0.97	0.01	0.01	4.39	0.04	0.05	16.86	0.16	0.18	40.45	0.37	0.42
AJD ΔF18590	8.7	39.85	0.37	0.42	0.93	0.01	0.01	4.49	0.04	0.05	14.37	0.13	0.15	45.27	0.42	0.47
AJD ΔA15906	7.57	38.90	0.36	0.41	0.79	0.01	0.01	4.31	0.04	0.04	13.00	0.12	0.14	44.00	0.41	0.46
AJD ΔD04092	8.74	36.08	0.33	0.38	0.99	0.01	0.01	4.98	0.05	0.05	14.95	0.14	0.16	42.05	0.39	0.44
AJD ΔC13508	9.56	37.33	0.35	0.39	0.97	0.01	0.01	4.81	0.04	0.05	17.91	0.17	0.19	43.11	0.40	0.45
AJD ΔB21780	9.54	37.19	0.35	0.39	1.06	0.01	0.01	4.10	0.04	0.04	16.88	0.16	0.18	42.35	0.39	0.44
AJD ΔE18348	8.2	38.40	0.36	0.40	0.95	0.01	0.01	4.84	0.04	0.05	14.81	0.14	0.15	44.19	0.41	0.46
D2	10	29.57	0.28	0.31	3.86	0.04	0.04	6.52	0.06	0.07	16.96	0.16	0.18	39.95	0.37	0.42
D3	8.2	30.70	0.28	0.32	3.62	0.03	0.04	7.02	0.06	0.07	17.95	0.17	0.19	41.33	0.38	0.43
D4	7.97	31.80	0.30	0.33	3.49	0.03	0.04	7.42	0.07	0.08	19.62	0.18	0.20	42.71	0.40	0.44
D5	8.27	30.93	0.29	0.32	3.67	0.03	0.04	6.78	0.06	0.07	18.07	0.17	0.19	41.39	0.39	0.43
D6	9.4	29.78	0.27	0.31	3.41	0.03	0.04	6.11	0.06	0.06	18.56	0.17	0.19	39.29	0.36	0.41
D7	8.94	9.30	0.11	0.10	8.43	0.10	0.09	7.81	0.09	0.08	14.21	0.16	0.15	25.54	0.29	0.27
D8	9.9	3.34	0.05	0.03	9.28	0.13	0.10	6.72	0.09	0.07	10.71	0.15	0.11	19.35	0.27	0.20

## Discussion

To maintain homeostasis under osmotic stress the yeasts may have developed multiple pathways of production of osmoprotectants. Unfavorable conditions, including high concentrations of salts, could lead to evolution of conserved cellular responses to osmotic stress. Erythrose reductases play a necessary and important role in the pathway of polyol synthesis. The response to osmotic stress in the yeast *Y. lipolytica* relies heavily on the production (impaired or functioning) of osmoprotectants, mainly erythritol. The importance of this process is indicated by the number of putative erythrose reductase genes in the genome as well as their sequence conservation in closely related *Yarrowia* species. The number of genes encoding putative erythrose reductases in species belonging to the *Yarrowia* clade suggests their significance and evolutionary maintenance mechanism.

It was observed that the influence of single gene knockouts on polyol production was very limited, suggesting that the function of one deleted gene might be undertaken by another ER homolog. That would also explain the abundance of homologs with high sequence conservation, as the entire process of osmoprotectant production is necessary for the yeast to survive under unfavorable, hypersaline conditions or temporary osmotic stress. Previously described analysis for *YALI0F18590g*^18^ and *YALI0C13508g*^31^ showed that the knockout strains of both of the genes were characterized by decreases in erythritol synthesis. The results of the present study are consistent for the *YALI0F18590g* knockout strain in the initial phase of the experiment and follow the trend for the *YALI0C13508g* strain. Previously reported knockout of *YALI0D07634g* improved erythritol synthesis with glucose as a carbon source^31^ and was not investigated in the experimental phase of our study due to the smaller percentage of similarity with the other putative ER. In the group ofanalyzed genes, there is an outlier, YALI0B07117p which is the reported YlER^30^. The lack of *YALI0B07117g* leads to the most significant drop in erythritol synthesis, which is consistent with the previously described results, as well as improvement of arabitol and mannitol production. Similar effects on polyols production are not observed for the rest of the analyzed single knockouts of *YALI0A15906g*, *YALI0B21780g*, *YALI0C09119g*, *YALID04092g* or *YALI0E18348g*.

Additionally, the analysis of mannitol and erythritol synthesis from glucose in the thermoresistant *Y. lipolytica* strain YALI-hsp90 revealed the significant increase of *YALI0B07117g* expression at higher temperatures. The increase of temperature during the experiments also led to improved production of mannitol from glucose. The ratio of mannitol to erythritol reached 2.07 at 35 °C as compared to 0.269 in the cultures grown at 30 °C. The expression of *YALI0C13508g* and *YALI0F18590g* did not increase in this study^36^. Those results further support the significant role of *YALIB07117g* in the polyol synthesis pathway. Moreover, *YALI0B07117g* is the only GCY gene conserved in all *Yarrowia* clade species including *Y. phangngaensis*, which has a single GCY gene. A possible explanation is a strong selective pressure acting on this gene to conserve its function in the genome. It is therefore of interest to understand its role in the metabolism of *Yarrowia* species. The evolutionary scenario of the ERs in the *Yarrowia* clade suggests that after the divergence of *C. hispaniensis*, which has a single copy of the gene belonging to GCY family, there were two successive duplications of the ancestral copy, leading to 3 in the number of ER in the ancestor of the *Yarrowia* genus (Fig. S3). The analysis reveals the possible sequence of events to be two duplications in the branch of *Y. alimentaria*, which has *YALI0D04092g, YALI0B07117g* and *YALI0B21780g* and two specific GCY genes. An additional duplication then occurred after the divergence of *Y. phangngaensis*, leading to *YALI0A15906g* and consequently to four ancestral copies for all other species. Specific evolutionary events occurred in specific branches, showing that the GCY gene family evolution is dynamic.

To analyze the role of selected erythrose reductases on polyol synthesis, the strain knocked-out for eight ER genes was obtained. The results gathered using this strain indicate the significant impact of impairment of the erythritol synthesis pathway on glycerol utilization under osmotic stress. However, the entire pathway of erythritol synthesis is still under debate, as there is a possible secondary pathway^37^. Glycerol utilization in the ESM was delayed for the D8 strain by 72 h as compared to the control strain. Similar results were not observed for the mannitol synthesis medium, which lacks sodium chloride and therefore induces lower osmotic pressure on the cells. The lack of 8 putative ER genes alters the polyol production and forces the production of arabitol and mannitol. Those polyols may act as a secondary defense to osmotic stress and the pathways of their synthesis may be intertwined with erythritol production, as was previously discussed^30,36^. It is a possible outcome as mannitol is produced in response to osmotic stress as one of the main mechanisms of protection by bacteria *Acinetobacter baumannii, Pseudomonas putida*, and *Acinetobacter baylyi*^38–40^.

The results obtained in this study indicate that sufficient production of erythritol in conditions with high osmotic pressure is maintained when six out of eight reductases analyzed in this study are functional in the cells. This is based on the polyol production profile of multiple knockout strains, which does not differ significantly between the D2 and D6 strains. The drop in erythritol synthesis and glycerol utilization is observed in D7 and D8 strains. Those strains also produce higher amounts of arabitol and mannitol than the control. Overall mannitol production decreases in favor of erythritol synthesis after all the glycerol in the medium is utilized. Based on the production profiles there is a strong possibility of the overshadowing of the impact of the deletion of *YALI0B07117g* on other YlERs in the multiple knockout strains.

Despite deletion of all eight erythrose reductase homologous genes, the production of erythritol was not abolished entirely. The decrease of erythritol production by 91% indicates the superior impact of this pathway on erythritol production. This result also suggests that alternative pathway or other genes responsible for erythritol production might be present, due to the significance of the process^37^. Therefore the need to knockout additional genes of proposed ER would have to be carried out.

The abundance of erythrose reductase genes may be an evolutionary mechanism allowing the yeast in the *Yarrowia* clade to inhabit hypersaline environments such as Guerrero Negro, Baja California Sur, and Mexico where the N-9 isolate of *Y. lipolytica* was discovered^41^. Similarly, other members of the *Yarrowia* clade possess the ability to inhabit marine environments, such as *C. hispaniensis*^42^ and *Yarrowia phangngaensis*^43^. Both of those organisms were tested for their ability to produce polyols in media containing high amounts of glycerol and glucose, as well as sodium chloride, to induce osmotic stress^27^. Surprisingly, those yeasts did not produce high amounts of polyols. On the contrary, *C. hispaniensis* did not produce significant amounts of erythritol in any of the tested conditions. However, *O. hispaniensis* and *Y. phangngensis* possess homologs of erythrose reductases in their genomes. Those results, combined with the limited, yet not completely blocked production of erythritol in the D8 strain of *Y. lipolytica*, raise the question whether the impaired polyol synthesis may also be the result of its accumulation in the cells. Both *C. hispaniensis* and *Y. phangngaensis* species possess only one member of the GCY group where other *Yarrowia* species have up to 5 genes. This may indicate the correlation between the number of YlERs belonging to this group and the ability to produce high amounts of polyols.

To possibly use the yeast *Y. lipolytica* to produce arabitol or mannitol from glycerol, the rest of the genes involved in erythritol production must be carefully knocked out, necessary not only to stop erythritol synthesis but also not to affect the production of arabitol and mannitol. Interestingly, the impairment of the erythritol synthesis pathway resulted in high productivity of citric acid in the media without addition of salt, which could be utilized, as citric acid is a value-added product of glycerol utilization. Further shift to arabitol production by *Y. lipolytica* could be possible according to the analysis of genes involved in mannitol synthesis by Wang et al. (2020)^17^. The authors showed that the knockout of the mannitol dehydrogenase gene (*YALI0D18964g*) disrupts synthesis of mannitol from glucose. All in all, the potential use of *Y. lipolytica* as a host for arabitol or mannitol production relies on a better understanding of the pathways leading to the synthesis of those compounds. The need to adapt and quickly respond to the unfavorable conditions in regards to osmotic stress could be a driving force behind the shift from erythritol synthesis to other polyols. The mechanism behind that change remains unknown. It is also a requirement to improve the growth parameters for the strains, as the long cultivation time may prove too expensive for industrial application.

## Conclusions

This study revealed the effects of different erythrose reductase homologs on polyol synthesis. Single and multiple knockouts were analyzed to specify the importance of each of the studied genes as well as the impact of several knockouts on the production of polyols. The obtained results indicate the key role of the *YALI0B07117g* gene on erythritol production in *Y. lipolytica*. This homolog is vital in maintaining the carbon flux towards erythritol. Erythritol synthesis is an important process in maintaining cell homeostasis and the proper response to osmotic stress. Due to the major role of this process, the yeast may have developed alternative pathways of erythritol synthesis. The lack of analyzed homologs of the erythrose reductase gene, which are characterized by high similarity, did not stop the process of erythritol synthesis completely. It might suggest the presence of other pathway(s) resulting in erythritol synthesis under stress conditions. Despite the presence of 8 highly homologous genes encoding the erythrose reductase enzyme, the lack of as many as 6 of them does not impair erythritol production significantly. This suggest their limited role in this pathway or a possible function in other pathways. The lack of the erythrose reductase genes impacted the erythritol synthesis and shifted the carbon flux towards other polyols, but the biomass was also at a high level. This may indicate at least partial flux towards that biomass production. In the conditions with lower osmotic pressure a large amount of citric acid was produced by the D8 strain. This feature could be more thoroughly examined and utilized. Further research should focus on alternative pathways of erythritol synthesis, and the possibility of stopping the process entirely would lead to obtaining a strain able to produce arabitol or mannitol from glycerol for industrial application.

## Materials and methods

### Strains, media and culture conditions

The bacterial and yeast strains used in this study are listed in Table 4.

**Table 4:** Strains of *E. coli* and *Y. lipolytica* used in this study.

Strain	Genotype or plasmid	Source
*E. coli*		
DH5α	F − endA1 glnV44 thi-1 recA1 relA1 gyrA96 deoR nupG Φ80dlacZΔM15 Δ(lacZYA-argF)U169, hsdR17(rK-mK+), λ–	^44^
DH5α	pUCURA	^45^
DH5α	Cre-lox	^45^
DH5α	pUCURA-B07117g	^30^
DH5α	pUCURA-F18590g	^18^
DH5α	pUCURA-C09119g	This study
DH5α	pUCURA-D04092g	This study
DH5α	pUCURA-A15906g	This study
DH5α	pUCURA-B21780g	This study
DH5α	pUCURA-C13508g	This study
DH5α	pUCURA-E18348g	This study
*Y. lipolytica*		
AJD	*MATA*, A101: ura3-302	^46^
AJD ΔB07117	*MATA*, A101: ura3-302 ΔB07117	^30^
AJD ΔF18590	*MATA*, A101: ura3-302 ΔF18590	^18^
AJD ΔC09119	*MATA*, A101: ura3-302 ΔC09119	This study
AJD ΔD04092	*MATA*, A101: ura3-302 ΔD04092	This study
AJD ΔA15906	*MATA*, A101: ura3-302 ΔA15906	This study
AJD ΔB21780	*MATA*, A101: ura3-302 ΔB21780	This study
AJD ΔC13508	*MATA*, A101: ura3-302 ΔC13508	This study
AJD ΔE18348	*MATA*, A101: ura3-302 ΔE18348	This study
D2	*MATA*, A101: ura3-302 ΔB07117 ΔC09119	This study
D3	*MATA*, A101: ura3-302 ΔB07117 ΔC09119 ΔF18590	This study
D4	*MATA*, A101: ura3-302 ΔB07117 ΔC09119 ΔF18590 ΔA15906	This study
D5	*MATA*, A101: ura3-302 ΔB07117 ΔC09119 ΔF18590 ΔA15906 ΔD04092	This study
D6	*MATA*, A101: ura3-302 ΔB07117 ΔC09119 ΔF18590 ΔA15906 ΔD04092 ΔC13508	This study
D7	*MATA*, A101: ura3-302 ΔB07117 ΔC09119 ΔF18590 ΔA15906 ΔD04092 ΔC13508 ΔB21780	This study
D8	*MATA*, A101: ura3-302 ΔB07117 ΔC09119 ΔF18590 ΔA15906 ΔD04092 ΔC13508 ΔB21780 ΔE18348	This study

*Escherichia coli* strains obtained in this study were grown in Luria Bertani medium (A&A Biotechnology, Gdańsk, Poland) at 37 °C. The supplementation of ampicillin (100 mg/L) was required to screen the transformants on media plates (LB medium with 2% agar).

*Y. lipolytica* strains were grown in YPD medium (A&A Biotechnology) containing 1% yeast extract and peptone and 2% glucose. This medium was used for obtaining inocula for shake flask experiments and growth analysis. The shake flask experiments were carried out in 0.25 L baffled flasks containing 0.03 L of erythritol synthesis medium (ESM) with 100 g/L of glycerol as the main carbon source, 2.3 g/L (NH_4_)_2_SO_4_, 1 g/L MgSO_4_ × 7H_2_O, 0.23 g/L KH_2_PO_4_, 1 g/L yeast extract (Merck, Germany) and NaCl 26.4 g/L. To obtain the stable pH throughout the experiment, 3 g/L of CaCO_3_ was added to the medium. The pH was adjusted to 3.0. The parameters of the cultivation were set at 28 °C and 200 rpm. The medium was prepared following the description by Mirończuk et al. (2017)^15^. The optical density (OD) at the beginning of the experiment was set at 0.3. The experiment was carried out for 96 h. After every 24 h, 300 µL of the sample was collected in sterile 1.5 mL Eppendorf tubes. The biomass was analyzed by filtering 10 mL of the medium after 96 h of cultivation on 0.45 µm pore membranes and drying at 120 °C 24 h after filtration. The growth analysis was performed in microplates with a final volume of 200 µL of the media. Additionally, mannitol synthesis medium (MSM) was used to analyze the osmotic stress response. In this experiment, the ESM medium was modified not to contain the sodium chloride.

### Cloning and transformation methodology

All the required restriction enzymes, T4 DNA ligase and Phusion high-fidelity DNA polymerase were purchased from Thermo Scientific (USA). The performed reactions followed standard protocols suggested by the manufacturer. Kits for genomic DNA extraction, Gel Out extraction and plasmid isolation were purchased from A&A Biotechnology (Poland). The procedures were performed following the protocols described by the supplier.

The transformation of *E. coli* was performed by the standard chemical protocol with selective media plates containing 100 mg/L of ampicillin. *Y. lipolytica* transformation to obtain the knockout mutants was carried out by the lithium acetate method, with YNB media plates supplemented with 2% glucose. The transformations resulted in eight single knockout mutants.

To obtain the multiple knockout mutants, the marker cassette had to be removed. To do so, the strain was transformed with the cre-lox plasmid and plated on a YPD medium plate with hygromycin (250 mg/L). Three days after the transformation the colonies were plated on a new YPD plate with hygromycin. This step was performed three times. Following that, colonies were plated on a YPD plate and a YNB plate with 2% glucose. The strains growing on the YPD medium and not on the YNB plate were selected for further confirmation of successful transformation. Cre-lox transformation was performed after each consecutive knockout. This procedure resulted in obtaining seven multiple knockout mutants (D2-D8).

### Genes disruption

The disruption of the erythrose reductase genes was obtained by the creation of disruption cassettes for each gene. Upstream regions were amplified by PCR with specific primers (gene name_Up_ F/R_enzyme name, for details see Supplementary Material, Table 1) from the genomic DNA of *Y. lipolytica*. Each fragment was then digested with restriction enzymes, as well as the pUC-Ura plasmid. Following the digestion and clean up, conducted using a clean-up kit (A&A Biotechnology, Poland), ligation and transformation to the *E. coli* competent cells was performed. Selection of transformants was carried out by PCR with primers used to create the upstream region for gene knockout. Following the selection, the correctly assembled vectors were isolated by plasmid mini isolation kit (A&A Biotechnology, Poland). This resulted in plasmids with the upstream region of the gene of interest. Next, the downstream region was amplified (gene name_Down_F/R_enzyme name) and digested, similarly to the upstream region. Each fragment was then cloned to the corresponding plasmid with upstream region integrated, resulting in a pΔgene_name vector. The second cloning followed the procedure for the upstream region. To obtain the disruption cassette, vectors were digested with restriction enzymes as stated in Up_F and Down_R primers for each erythrose reductase gene and used for lithium acetate transformation of the yeast *Y. lipolytica*. To confirm the proper integration of the knockout cassette the genomic DNA of the mutants was extracted, and PCR analysis was performed. All the used primers are listed in Table 1 in Supplementary data.

The procedure for a gene knockout of an exemplar ER gene followed the described above procedure. Amplification of the upstream region with primers YALI0D04092_up_F_HindIII and YALI0D04092_up_R_ApaI based on the genomic DNA of *Y. lipolytica* WT strain. Enzymatic digestion of the obtained PCR product and pUC_Ura plasmid with enzymes HindIII and ApaI. Ligation of the digested PCR product and vector and transformation to the competent *E.coli* strain. Selection of vectors with integrated upstream region and plasmid isolation. Amplification of the downstream region of the gene with primers YALI0D04092_down_F_NotI and YALI0D04092_down_R_MssI. Digestion of the plasmid with upstream region of the gene and the downstream PCR product with enzymes NotI and MssI. Ligation of the fragment and vector and transformation to *E. coli* competent cells. Selection of transformants with integrated downstream region to the vector and plasmid isolation. Digestion of the correctly assembled plasmid with enzyme HindIII and MssI to obtain the disruption cassette, that can be used to transform *Y. lipolytica*.

### Bioscreen C analysis

All the obtained strains and the WT strain were grown in YNB medium with 2% glucose in 0.1 L Erlenmeyer flasks with a working volume of 0.01 L for 48 h. 1 mL of each culture was additionally transferred to new flasks containing 0.01 L of sterile YNB medium with 2% glucose for 48 h to deplete the cells from all the stored nutrients. The growth analysis was performed in 100-well plates containing 200 µL of YPD medium, YNB medium supplemented with 5% glucose or glycerol or 10% glycerol. The cells from the cultures were centrifuged and washed with Milli-Q water. Each well was then inoculated to an OD_600_ of 0.1. The Bioscreen C system (Oy Growth Curves Ab Ltd., Helsinki, Finland) was used to conduct the study. The culture parameters were set at 28 °C under continuous agitation, with a pause of 5 s before the measurement. Growth of the strains was monitored by measuring the optical density at the range of 420–560 nm every 30 min for 72 h. The analysis was performed in triplicate.

### Analytical methods

The samples obtained from the shake flask experiments were centrifuged for 15 min at 15,000 rpm. Supernatants were then transferred to new Eppendorf tubes and centrifuged again for 10 min at 15,000 rpm. To characterize the concentration of polyols (erythritol, arabitol and mannitol), glycerol and citric acid, the centrifuged samples were diluted 10 times and analyzed by HPLC analysis using a HyperRez Carbohydrate H + column (Thermo Scientific), coupled to a UV detector (λ = 210 nm) (Dionex, USA) and a refractive index detector (Shodex, Japan). Elution of the column was performed with 25 mM trifluoroacetic acid at 65 °C with a flow rate of 600 µL/min.

### Calculation of productivity and yield

To calculate the yield and productivity in the shake flask experiments, the following formulas were used as described by Rywińska et al. (2015)^47^:

$${\text{Y}} = { }\frac{{\text{P}}}{{\text{S}}}\quad {\text{Q}} = \frac{{\text{P}}}{{\text{t}}}$$

Y—yield, expressed in g g^-1^; P—amount of product in g/dm^-3^; S—amount of substrate in g/dm^-3^; Q—productivity, expressed in g dm^−3^ h^−1^; time—time at the end of the experiment in h.

### Bioinformatic analyses

Homologues of 10 *Y. lipolytica* proteins of the aldo/keto reductase family were searched in the *Yarrowia* genomes previously published in Červenák and colleagues by sequence homology^48^. The blastp threshold for E-value was set to 1.e-20. Protein sequences were aligned using muscle in the multi-platform graphical user interface Seaview version 4.6.2^49^. Then, Gblocks was used to select blocks of conserved sites (268 sites) useful for phylogenetic analysis^50^. A distance method based tree was constructed with BioNJ and a Poisson distance^51^. Investigation of the GCY group of proteins was done by synteny analysis, phylogeny and reconstruction of the evolutionary scenario. Altogether, the GCY group includes 43 proteins, one from *C. hispaniensis* and 42 from *Yarrowia* species. The protein sequences were aligned with muscle (Seaview platform) and cleaned with Gblocks. A phylogenetic tree based on the resulting alignment of 302 sites was reconstructed by maximum likelihood using PhyML 3.0 with a LG evolutionary model^52^. Robustness of the tree was assessed by the approximate likelihood ratio test approach (aLRT). Finally, to reconstruct the evolutionary scenario of the GCY gene family, synteny conservation was investigated by checking manually the *Y. lipolytica* homologues of three genes flanking GCY genes on each DNA strand using artemis genome browser^53^.

## Supplementary Information

Supplementary Information.

## Data Availability

The authors promise the availability of supporting data.aleksandra.mironczuk@upwr.edu.pl.
