# Supplementary material for: In-depth analysis of erythrose reductase homologs in Yarrowia lipolytica
Source: Sci Rep. 2023 Jun 5;13:9129. doi: 10.1038/s41598-023-36152-x (PMC10241868; doi:10.1038/s41598-023-36152-x)
Supplement: Supplementary file 1 — Supplementary Information. [file 41598_2023_36152_MOESM1_ESM.pdf]

Supplementary data

In-depth analysis of erythrose reductase homologs in *Yarrowia lipolytica*

Mateusz Szczepańczyk[1], Dorota A. Rzechonek[1], , Cécile Neuvéglise[2], Aleksandra M. Mirończuk*

[1]Wrocław University of Environmental and Life Sciences, Institute of Environmental Biology, Laboratory for Biosustainability, 5b Kozuchowska St., 51-631, Wroclaw, Poland

[2] INRAE, Institut Agro, SPO, University Montpellier, 34060 Montpellier, France

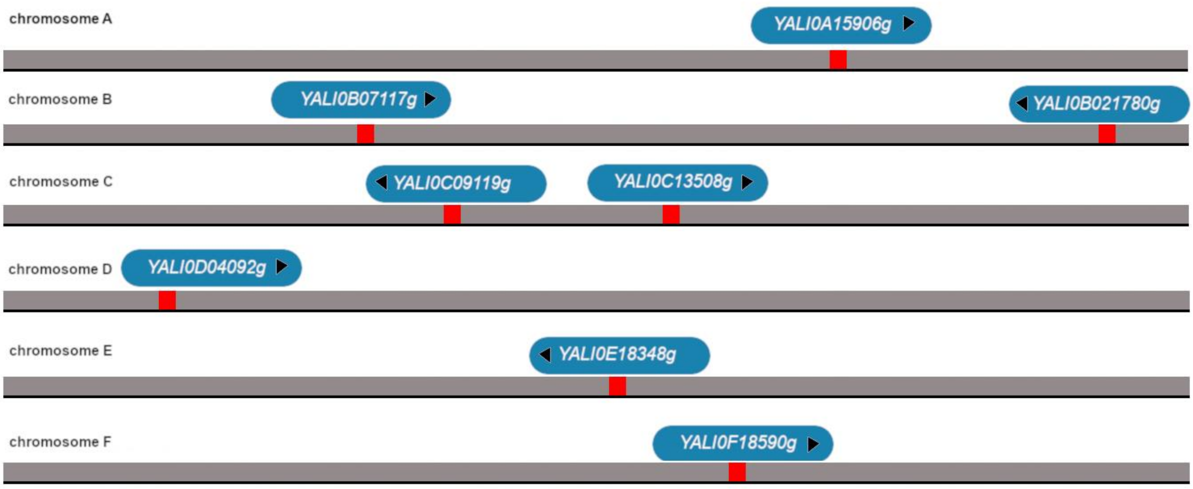

Figure 1. Graphical representation of the genomic location of all the analyzed erythrose reductase genes in the genome of *Yarrowia lipolytica* CLIB122. Figures representing chromosomes not scaled to their lengths. Red brackets indicate the region on the chromosome the erythrose reductase is located in. The teal markers represent the gene of erythrose reductase present on each of the chromosomes with the indication of location on the sense or anti-sense strand, as displayed by triangles.

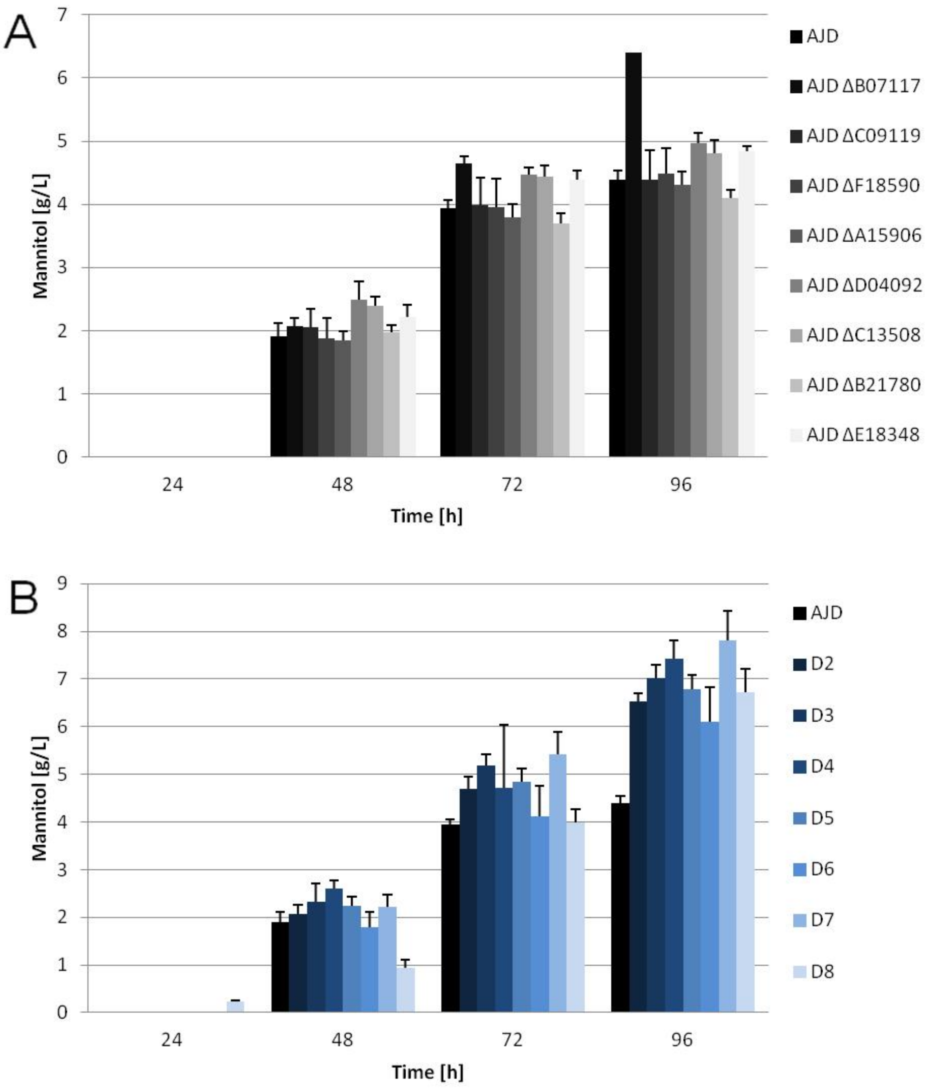

Figure 2. Mannitol synthesis in erythritol synthesis medium for single gene knockouts (A) and multiple YlER knockout (B) with AJD strain as a control in both panels (black).

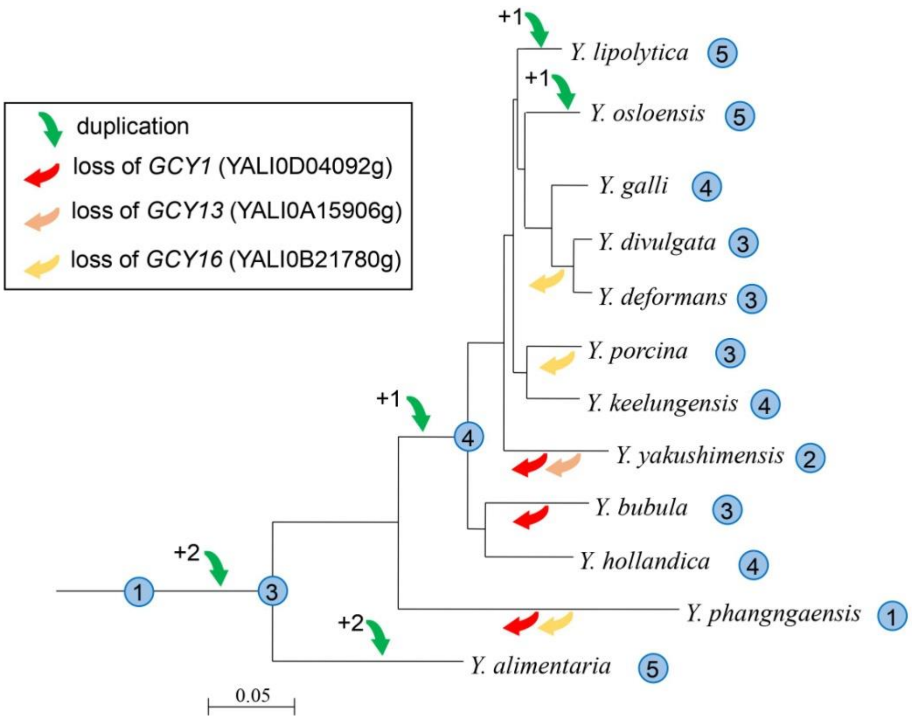

Figure 3. Reconstruction of an evolutionary scenario of GCY genes in *Yarrowia* clade species. Green arrows represent duplication events and red, orange and yellow the loss of *GCY1, GCY13* and *GCY16*, respectively. The phylogenetic tree is based on the concatenation 97 protein family alignments.

Table 1. Primers used in this study.

Primer	Sequence (5′ -> 3′)	Aim
YALI0C09119_up_F_BamHI	CGAGGATCCCAAGTCCGTCGAGAAG	Knock-out of the *YALI0C09119g* gene
YALI0C09119_up_R_ISceI	CGCCCATTACCCTGTTATCCCTACGGGTTTGAACCTGAAG	
YALI0C09119_down_F_ISceI	CGCTAGGGATAACAGGGTAATGAAACGCCATGTCGATGTC	
YALI0C091197_down_R_KpnI	CGAGGTACCACGACGTCCTCCAAAG	
YALI0D04092_up_F_HindIII	GCGAAGCTTGAACGGGTACGTTGAC	Knock-out of the *YALI0D04092g* gene
YALI0D04092_up_R_ApaI	TATGGGCCCTCTTGCCGTTGTTCATAG	
YALI0D04092_down_F_NotI	CGTGCGGCCGCGATACAGATATTTATTAG	
YALI0D04092_down_R_MssI	CGGGTTTAAACGCAAACAGGCGTTGTC	
Test- D04092_F	CGGGATGCAGTTGTTGAGGAGAG	Verification of the knock-out
Test- D04092_R	GCTCCACGTGCTCGGTAATG	

YALI0A15906_up_F_HindIII	GCAAGCTTATGGGAGTCGGAGATCATTC	Knock-out of the *YALI0A15906g* gene
YALI0A15906_up_R_PaeI	ATGCATGCTCTTGTTGTGGTTGTGATTG	
YALI0A15906_down_F_NotI	ATGCGGCCGCTTACAGGATGAGAGTGTGTATG	
YALI0A15906_down_R_MssI	ATGTTTAAACTTTTGAGCTCCTTGGCGATCT	
Test-A15906_F	TTCCACTCGGGATGGCATTC	Verification of the knock-out
Test-A15906_R	CGGACTCTCCAGACACAAAC	
YALI0B21780_up_F_SalI	ATGTCGACGTATGACCGTCGTTGGCAAGTG	Knock-out of the *YALI0B21780g* gene
YALI0B21780_up_R_ApaI	ATGGGCCCACATAACATAGACCTTAGAAATTG	
YALI0B21780_down_F_NotI	ATGCGGCCGCAACCACAGCATGGAAAATAGC	
YALI0B21780_down_R_MssI	ATGTTTAAACGAGAAGGGGCACTTGAACAC	
Test-B21780_F	GGCCAGTGAGCTCTGAAACG	Verification of the knock-out
Test-B21780_R	GGGCTTATGGATGGAACACTTG	
YALI0C13508_up_F_HindIII	GCAAGCTTAATGGCGAACGAATCGCTACAG	Knock-out of the *YALI0C13508g* gene
YALI0C13508_up_R_SalI	ATGTCGACGCGTGGGTACAAGTGCAGTGTAG	
YALI0C13508_down_F_NotI	ATGCGGCCGCTCAGTGACCTGATCGAATTG	
YALI0C13508_down_R_MssI	ATGTTTAAACCAACAAAGGTGAGGAAGAAG	
Test-C13508_F	TAGTGGCGTCTTCCGTAAGC	Verification of the knock-out
Test-C13508_R	ACAGAGGCAGTTGGAGTGAG	
YALI0E18348_up_F_HindIII	ATAAGCTTGGTGTCGGGAAGCCATGCAGTC	Knock-out of the *YALI0E18348g* gene
YALI0E18348_up_R_ApaI	ATGGGCCCTTACTAGACATTTGAGCGTGAGAG	
YALI0E18348_down_F_NotI	ATGCGGCCGCCAGACTCCAGGGGGGATCAAC	
YALI0E18348_down_R_MssI	ATGTTTAAACGCCGAGCAGACGGACGATTTGG	
Test-E18348_F	AATGGATCTTGCCACAGTTC	Verification of the knock-out
Test-E18348_R	GGAGCAGCAGAATGAGAAGC	
YALI0B07117_up_F_HindIII	CGTAAGCTTTACACTCCCGCACAAAC	Knock-out of the *YALI0B07117g* gene
YALI0B07117_up_R_SalI	CGTGTCGACGGTCTTGCTCGGATTC	
YALI0B07117_down_F_NotI	TCAGCGGCCGCAGCTTGGTGAACCATATTT	
YALI0B07117_down_R_SacII	TATCCGCGGGCTCCAGACGAGTAAATC	
Test-B07117_F	CCCGGTTTATTGACCTCCTTACAGC	Verification of the knock-out

Test-B7117_R	CGGAACTTCTGTTTCTGCCATCTGAC	
URA_col_F	GGTACTGGTGCTTGACAGTG	Verification of the knock-outs
URA_col_R	CTCGAGCTAACGTCCACAAG	
